# Editorial: Non-destructive quality assessment and intelligent packaging of agricultural products

**DOI:** 10.3389/fpls.2026.1820532

**Published:** 2026-03-19

**Authors:** Jiangbo Li

**Affiliations:** Intelligent Equipment Research Center, Beijing Academy of Agriculture and Forestry Sciences, Beijing, China

**Keywords:** machine vision technology, model construction, near-infrared spectroscopy (NIR), non-destructive testing, quality assessment (IQA)

Agricultural products, as the starting point of the food production chain, their quality and safety are of paramount importance to the national economy and people’s livelihood. From the fields to the dining tables of the common people, every aspect of safety control directly affects the physical health of consumers. High-quality and safe agricultural products can provide the human body with sufficient and healthy nutrition, reduce the risk of foodborne diseases, and safeguard people’s happy lives. At the same time, the quality and safety of agricultural products is also the core support for the sustainable development of agriculture. In the globalized market, only by strictly controlling quality can the international competitiveness of agricultural products be enhanced, thereby further promoting the virtuous cycle of the agricultural industry.

In the quality inspection of agricultural products, the near-infrared spectroscopy and machine vision technology are the two most commonly used and crucial non-destructive testing techniques. The near-infrared spectroscopy technology analyzes the internal components of agricultural products without damaging them, enabling rapid and accurate quality assessment and significantly improving the efficiency and reliability of the inspection. The machine vision technology, by leveraging image acquisition and AI algorithms, efficiently identifies surface defects, pests, and diseases, reducing human errors and ensuring product safety. This research topic focuses on the application of these two technologies in the detection of the quality of some agricultural products, such as tomato, potato, rice and tea leaf.

Soluble solids content (SSC) is an important indicator for evaluating tomato flavor, and conventional physical and chemical methods are both time-consuming and destructive. Cai et al. used full transmission visible-near infrared spectroscopy technique for non-destructive detection of soluble solids content in tomatoes. A combination wavelength selection method (i.e. backward variable selection-partial least squares and simulated annealing (BVS-PLS and SA)) was used to select 20 wavelengths from the full 2048 spectral data. Based on these selected wavelengths, a partial least squares regression model was constructed, which obtained better prediction results (R_p_ = 0.912, RMSEP = 0.354%) compared to the full spectrum model. This study demonstrates the effectiveness of visible-near infrared spectroscopy in predicting tomato SSC. Blackheart disease is one of the most common physiological diseases of potatoes during storage. In the early stage, this disease cannot be detected by its appearance. If not identified and removed in time, this disease will seriously damage the quality of the entire batch of potatoes and even cause food safety issues. Guo et al. used visible-near infrared (Vis/NIR) spectroscopy to conduct online analysis on potatoes with varying degrees of blackheart. They developed an efficient and lightweight detection mode by introducing the depthwise convolution, pointwise convolution, and efficient channel attention modules into the ResNet model. The study found that the prediction accuracy for blackheart and healthy potatoes test sets reached 0.971 using the original spectrum combined with a modified ResNet model, and also, the modified ResNet model significantly reduced the number of parameters, achieving a substantial 62.71% reduction in model complexity. The protein content in rice is an important determinant of its unique structure, physical properties and nutritional characteristics. The traditional method for determining the protein content of rice-chemical analysis - requires a significant amount of manpower, time and cost. Yang et al. attempted to use near-infrared spectroscopy and deep learning technology to conduct non-destructive detection of the protein content of rice with husks (paddy rice). Three models, namely Partial Least Squares Regression, Support Vector Regression, and Deep Neural Network (DNN), were constructed and compared. The results showed that the DNN model was the best. Therefore, they concluded that near-infrared spectroscopy technology has commercial feasibility for non-destructive prediction of protein content protein content of rice with husks. In addition to near-infrared spectroscopy, machine vision, as another important non-destructive detection technology, was successfully used by Zhan et al. to detect diseases and defects in tea leaves. The efficiency of detecting tea leaf diseases and defects directly affects the quality and yield of tea. In actual production, inspectors may encounter problems such as lack of experience and fatigue, which leads to incomplete and slow detection results. Combined with machine vision technique, Zhan et al. developed the BHC-YOLO model for detecting defects and diseases of tea leaves. Compared with the current mainstream models, the confidence level increased by 7.1%, mAP0.5 increased by 8%, and reached 94.5%.

These studies provide reference for relevant scholars to promote the application of near-infrared spectroscopy and machine vision technologies in this field. The comprehensive application of these technologies not only ensures consumer health, but also promotes the process of agricultural intelligence and standardization, building a solid technological defense line for food quality and safety.

